# Supplementary material for: Chromothripsis during telomere crisis is independent of NHEJ, and consistent with a replicative origin
Source: Genome Res. 2019 May;29(5):737–49. doi: 10.1101/gr.240705.118 (PMC6499312; doi:10.1101/gr.240705.118)
Supplement: Supplemental Material [file supp_gr.240705.118_Supplemental_file_1.zip › contigs/annotated_contigs/DB96/contig.2.DB96_length_490_mean_cov_23.3448979592.docx]

**DB96_length_490_mean_cov_23.3448979592**

```
TAAAGAAATAATCCTTGTGGGAAGGAAAACAAATACATATAGGAATAAAGTATGGGCTGGGCATGGTGCCTCACACCTGTAATCCCAGC
 >chr1:68256317-68256650 - E=8e-162
ACTTTGAGAGGCCAAGGCGGGTAGATCACGAGGTCAGGAGTTGGAGATCAGCCTGACAAACATGGTGAAACCTTGTCTCTACTAAAAAT

ACAAAAATTAGCCAGACGTGGTGGTGGCGGCACACGCCTGTAATCCCAGCTACTCAGGAGGCTGAGGCAGGAGAATCGCTTGAACGCGG

GAGGTGGAGGTTGCGGTGAGCCGAGATGGTGCCACTGCACTCCAGCCTGAGCAACAGAGCGAGATT|AGTTTTCTTGAGAAAGCTAAAT
                                                                    >chr1:68280983-68281
CTGAGGATTCTGGGACATGAGTGTTACTCATGGCCAGCGTCATCATACGGAGCCTGGGAGCAAATTGTTTTTTCAAGAGGTGTATGTAG
140 - E=7e-80
GCTGTTGAATTTTGACTAGAAATGGCTTTAGTGGTCAACATTAATT
```
